# Effect of mydriasis on macular and peripapillary metrics in swept-source optical coherence tomography angiography

**DOI:** 10.3389/fendo.2024.1292255

**Published:** 2024-02-28

**Authors:** Feng Zhang, Ying’an Li, Zijing Du, Hong Sun, Lijie Xie, Yingying Liang, Siwen Zang, Wei Sun, Honghua Yu, Yijun Hu

**Affiliations:** ^1^ Guangdong Eye Institute, Department of Ophthalmology, Guangdong Provincial People’s Hospital (Guangdong Academy of Medical Sciences), Southern Medical University, Guangzhou, China; ^2^ Department of Ophthalmology, Linyi People’s Hospital, Linyi, Shandong, China; ^3^ Department of Ophthalmology, The First People’s Hospital of Tancheng, Linyi, Shandong, China; ^4^ Guangdong Provincial Key Laboratory of Artificial Intelligence in Medical Image Analysis and Application, Guangdong Provincial People’s Hospital, Guangdong Academy of Medical Sciences, Guangzhou, China

**Keywords:** swept-source optical coherence tomography angiography (SS-OCTA), mydriasis, retinal vessel density, retinal thickness, ocular

## Abstract

**Introduction:**

The study aimed to evaluate the effect of mydriasis on macular and peripapillary metrics with swept-source optical coherence tomography angiography (SS-OCTA) in healthy subjects.

**Methods:**

Thirty-five healthy subjects were included. The macular region was scanned by the 3×3mm mode and 6×6mm mode, and the peripapillary region was scanned by the 4.5×4.5mm mode on both eyes with SS-OCTA before and after mydriasis. Macular and peripapillary metrics, including retinal vessel density (VD) and fundus thickness were measured by the built-in program. Data of the right eye were analyzed.

**Results:**

The signal strength of the scans was comparable before and after mydriasis (all *P*>0.05). There were no significant differences in foveal avascular zone (FAZ) parameters and retinal VD of most sectors in both macular and peripapillary areas (all *P*>0.05). Choroidal thickness was decreased, outer and whole retinal thickness was increased in most of the macular sectors after mydriasis (all *P*<0.05). Choroidal thickness was decreased in all the peripapillary sectors, but whole retinal thickness and GCC thickness were increased in some peripapillary sectors after mydriasis (all *P*<0.05).

**Conclusions:**

FAZ parameters and retinal VD in the most macular and peripapillary regions are not affected by mydriasis. The thickness of the choroid is decreased after mydriasis, while the thickness of retinal layers in some sectors may be increased after mydriasis.

## 1 Introduction

Optical coherence tomography angiography (OCTA) is a non-invasive and three-dimensional imaging technology to observe retinal and choroidal microcirculation *in vivo*. The utilization of OCTA proved beneficial for assessing retinal vascular changes in diabetic macular edema (DME) and monitoring the progress of therapeutic interventions (1, 2). The first-generation OCTA is spectrum-domain OCTA (SD-OCTA) with advantages over fluorescein angiography, such as fast, dye-free, depth-resolved visualization of retinal vessels, and classy representation of microvascular information (3). The advent of swept-source OCTA (SS-OCTA), the latest OCTA technology, can provide clinicians with retinal and choroidal images with more details and less time in scanning. Using a laser light source with a longer wavelength, the high resolution of SS-OCTA allows the scanning of larger areas and deeper tissue in one single high-definition widefield image (4). However, OCTA metrics, including vessel density and thickness of different retinal layers, may be affected by the size of the pupil (5, 6), and media opacities (7).

Although mydriasis is usually performed before OCTA examination in clinical routine, it may be time-consuming, causing blurred vision and acute angle closure (especially in Southeast Asian) (8). Especially for diabetic patients, it takes a long time for full mydriasis, due to degeneration of the nerve that controls the ciliary muscle (9). Therefore, it would be meaningful to evaluate the effect of mydriasis on retinal and choroidal metrics of OCTA. So far, a few studies have investigated the effects of mydriasis on SD-OCTA parameters with inconsistent results. According to previous studies, mydriasis using the combination of 0.5% tropicamide and 0.5% phenylephrine resulted in a significant reduction of peripapillary vessel density (VD), but not macular VD on SD-OCTA (6). In contrary, neither mydriasis by 1% tropicamide nor mydriasis by 2.5% phenylephrine significantly affected macular and peripapillary microcirculation with SD-OCTA imaging (10). The effect of mydriasis on retinal and choroidal thickness seemed to be different. Retinal nerve fiber layer (RNFL) and ganglion cell complex (GCC) thickness were not changed significantly after pupil dilation (11), while choroidal thickness was reduced by the combination of 0.5% tropicamide and 0.5% phenylephrine hydrochloride (5).

To date, the potential effect of mydriasis on SS-OCTA parameters is still unknown. More importantly, the agreement of these parameters before and after mydriasis also need to be investigated. Thus, the current study aimed to evaluate the difference and agreement of macular and peripapillary metrics with SS-OCTA in healthy subjects before and after mydriasis.

## 2 Methods

### 2.1 Participants

This prospective, observational study was performed in compliance with the Declaration of Helsinki and approved by the Research Ethics Committee of Guangdong General Hospital (Approval number: KY-Q-2022-341-03). Informed consent was obtained from all participants before entering the study. Thirty-five individuals without retinal diseases or media opacities were recruited for the study, between August and September 2022.

To calculate the sample size, the formula n ≥ log(1-β)/log(1-α) was used. Here, n represents the sample size, α is the discordance rate, and β is the tolerance probability. Assuming α=0.05 and β=80%, the calculated value of n was 32 (12).

### 2.2 Inclusion and exclusion criteria

For inclusion, all participants had to have best-corrected visual acuity (BCVA) better than 0.8 (decimal), refractive error less than ± 2.0 diopters, an intraocular pressure (IOP) less than 21 mmHg, and no history of ocular surgery or any retinal/optic nerve disease. Exclusion criteria are significant media opacities (e.g., dense cataract), any medication that could affect the circulation, history of smoking and alcohol consumption, and signal strength index < 8 (e.g., severe dry eye, small pupils within 2.5mm, or excessive eye motion, etc.).

### 2.3 Examination procedure

All participants underwent a comprehensive ophthalmologic examination, including BCVA (decimal, converted to LogMAR in analysis), IOP measurement (mmHg), refraction, slit-lamp biomicroscopy, indirect ophthalmoscopy, and SS-OCTA (VG200D; SVision Imaging, Henan, China).

After the estimation of IOP and slit-lamp examination, both eyes of all subjects were imaged with SS-OCTA system before and after mydriasis. Compound tropicamide eye drop, which contained tropicamide (0.5%) and phenylephrine hydrochloride (0.5%), was administered and repeated three times with an interval of 10 minutes to fully dilate the pupils. This procedure of mydriasis between two SS-OCTA scanning sessions took about 30 minutes for every individual. For each subject, only the data of right eye with a signal strength index ≥ 8 was included in the analysis.

The commercial OCTA device is equipped with a 1050 nm wavelength swept-source laser (990-1100 nm full width) and a scan rate of 200,000 A-scans per second. This device performs a full width at half maximum axial resolution of about 5μm in tissue, the lateral resolution of 15μm at the retinal surface, and a scan depth of approximately 3μm. This system contains an eye-tracking utility based on an integrated confocal scanning laser ophthalmoscope to eliminate eye-motion artifacts. For OCTA scans, images were obtained with a raster scan protocol of 512 horizontal B-scans using follow-up mode to ensure the same location as the baseline measurement before mydriasis. To avoid the segmentation error, manual correction was performed in the procedure of OCTA imaging (13).

**Figure 1:**
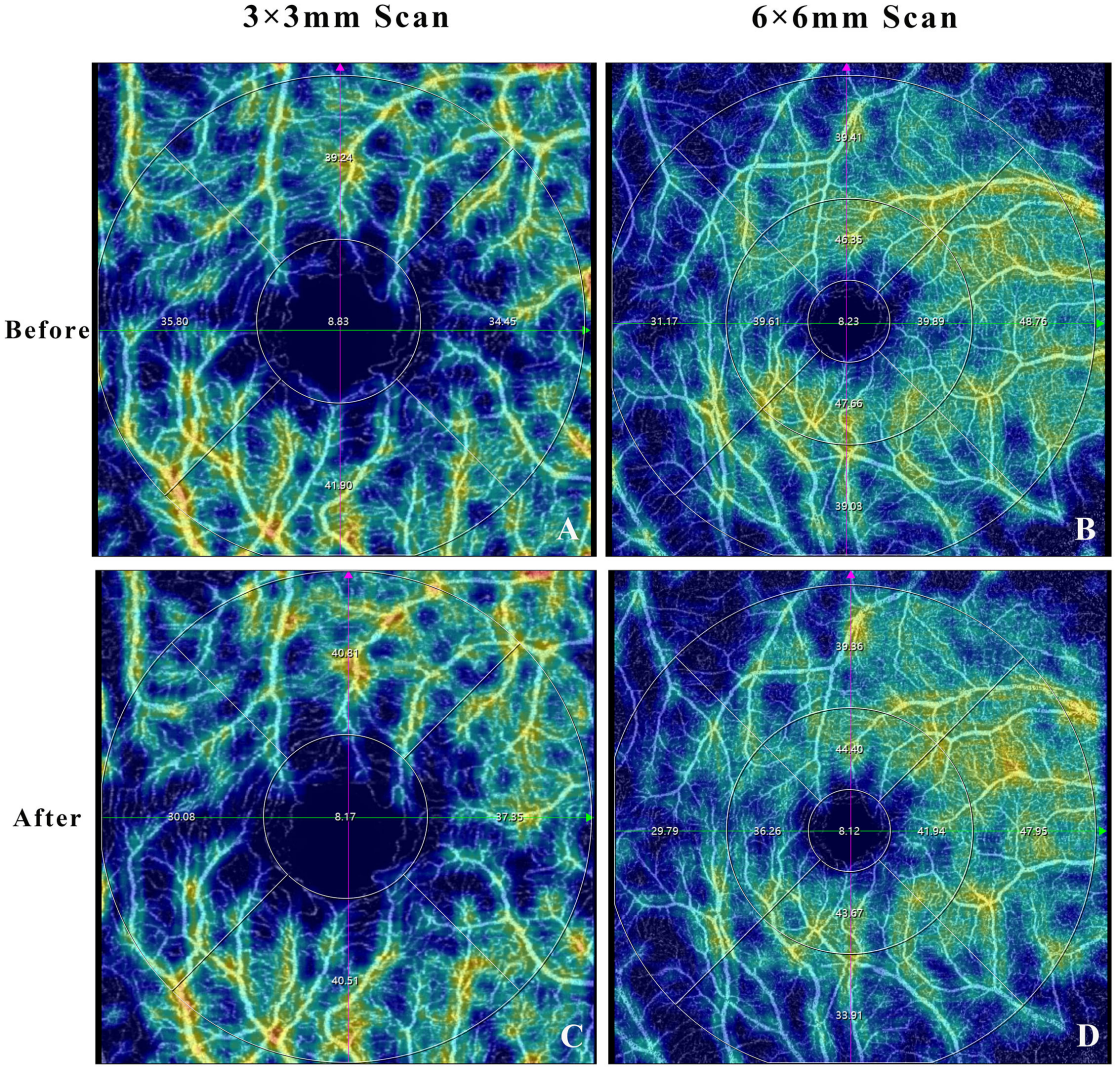
Optical coherence tomography angiography image showing the example of macular superficial vascular plexus before **(A, B)** and after **(C, D)** mydriasis.

**Figure 2:**
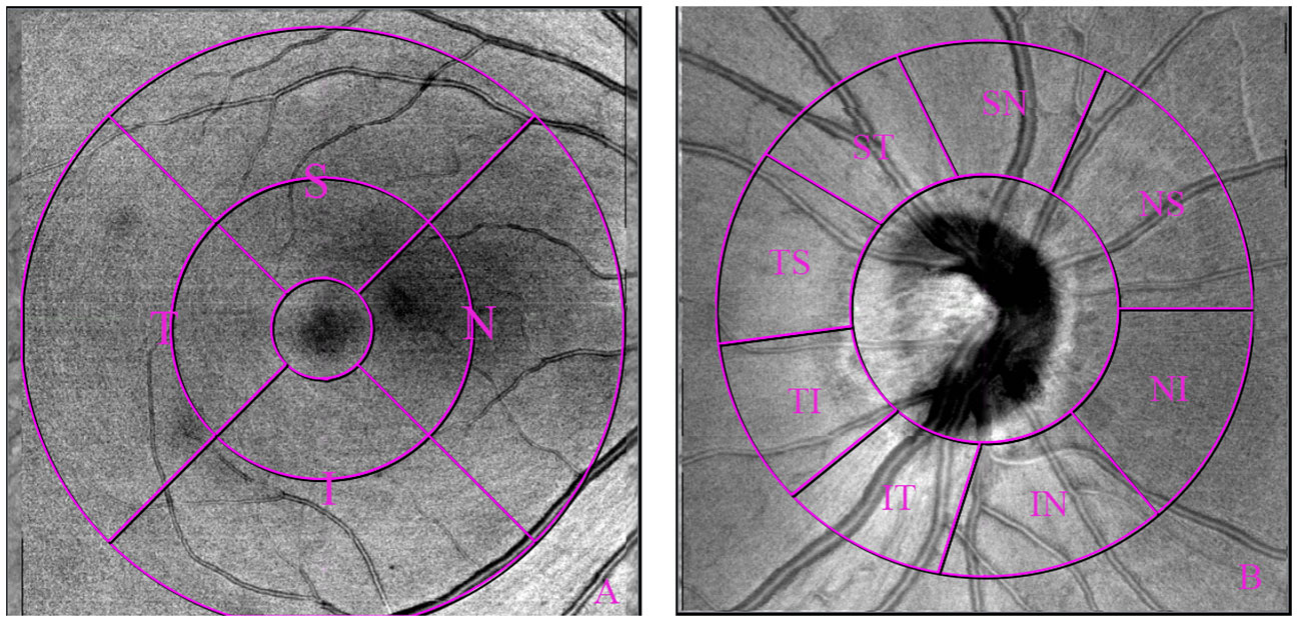
Optical coherence tomography angiography 6×6mm macular scan image and 4.5×4.5mm optic nerve scan image showing region of interest measured: macular [subdivided into 1-3mm and 3-6mm annulus, **(A)**], and peripapillary region [subdivided into 8 sectors, **(B)**].

Macular 3 × 3mm and 6 × 6mm scans, as well as optic nerve 4.5 *×* 4.5mm scan were acquired using the commercial SS-OCTA system by the same experienced investigator (Feng Zhang). For macular scans, superficial vascular plexus (SVP), intermediate capillary plexus (ICP), and deep capillary plexus (DCP) were measured by built-in algorithms (**Figure 1**). For peripapillary scans, vessel density in 2-4mm annular area was measured. For all scans, the thickness of choroid, outer retina, whole retina, retinal nerve fiber layer (RNFL), and the ganglion cell complex (GCC) was obtained with built-in algorithms. In the Macular 3 × 3mm images, foveal avascular zone (FAZ) parameters were detected, including FAZ area, perimeter, circularity index (CI), and foveal vessel density in 300μm area (FD-300). Following the Early Treatment Diabetic Retinopathy Study (ETDRS) partition, fractal dimensions were detected in 1-3mm and 3-6mm annulus area (i.e., S, superior; T, temporal; I, inferior; N, nasal) (**Figure 2**). In the optic nerve 4.5 *×* 4.5mm scans, the 2-4mm annular area was delineated into eight sectors by a built-in algorithm automatically (i.e., NS, nasosuperior; NI, nasoinferior; IN, inferonasal; IT, inferotemporal; TI, temporoinferior; TS, temporosuperior; ST, superotemporal; SN, superonasal) (**Figure 2**).

### 2.4 Data analysis

The data were given as the mean ± SD for normally distributed data and median with 25th and 75th quartiles for skewed data. Categorical data was shown as frequency and percent (n; %). The normality of the data was evaluated by the Shapiro - Wilk test. Wilcoxon sign-rank tests or paired *t*-test was used to assess the differences between before and after mydriasis. The agreement of OCTA parameters before and after mydriasis was assessed using intraclass correlation (ICC), and 95% limits of agreement (LoA) on Bland-Altman plots. A *P* < 0.05 was considered statistically significant. Statistical analysis was performed in IBM *SPSS* Statistics software version 23.0 (IBM Corp., Armonk, NY, USA) and *Prism* software (GraphPad *Prism*, *Prism* 8, version 8.0.2).

## 3 Results

Thirty-five right eyes of 35 healthy subjects were included in this study (16 males, 19 females), with a median age of 26 years (range from 18 to 68 years) (**Supplementary Table 1**). The median BCVA was 0.00 (0.00 - 0.00) logMAR. The signal strength of the three scan modes was comparable before and after mydriasis (all *P* > 0.05) (**Supplementary Table 2**).

### 3.1 Comparison of retinal VD before and after mydriasis.

For macular 3×3mm scan mode, FAZ parameters and VD in 1-3mm annular area were compared before and after mydriasis (**Tables 1**, **2**). There was no significant difference in FAZ parameters, including FAZ area, perimeter, CI, and FD-300 (*P*=0.077-0.851). There was no significant difference in macular VD in the whole annular area and four quadrants of SVP (*P*=0.264-0.841), ICP (*P*=0.452-0.964), and DCP (*P*=0.094-0.824), except SVP in inferior quadrant (*P*=0.023) and DCP in nasal quadrant (*P*=0.022).

**Table 1:** FAZ parameters of macular 3×3mm scan before and after mydriasis.

Parameter	Before mydriasis	After mydriasis	Difference	P value
FAZ area (mm2)	0.34 ± 0.13	0.34 ± 0.13	0.00 ± 0.01	0.851*
Perimeter (mm)	2.35 ± 0.47	2.40 ± 0.43	-0.05 ± 0.16	0.116*
CI	0.74 ± 0.08	0.71 ± 0.09	0.03 ± 0.08	0.077*
FD-300 (%)	38.75 ± 3.62	39.28 ± 3.75	-0.53 ± 2.63	0.238^†^

FAZ, Foveal avascular zone; CI, Circularity index; FD-300, Foveal vessel density in 300μm area. Values are presented as mean ± SD. Values were compared by ^*^Wilcoxon sign-rank tests or ^†^paired t-test.

**Table 2:** Macular vessel density of superficial vascular plexus (SVP), intermediate capillary plexus (ICP), and deep capillary plexus (DCP) in 1-3mm annular area of macular 3×3mm scan before and after mydriasis.

Area	Before mydriasis (%)	After mydriasis (%)	Difference (%)	*P* value^†^
**SVP- Whole**	**40.30 ± 3.55**	**40.70 ± 4.10**	**-0.40 ± 2.07**	**0.264**
SVP-S	44.91 ± 4.68	44.83 ± 4.66	0.08 ± 2.25	0.841
SVP-T	34.03 ± 3.86	34.57 ± 4.41	-0.54 ± 3.27	0.337
SVP-I	42.71 ± 3.97	43.66 ± 4.75	-0.95 ± 2.36	*0.023*
SVP-N	39.59 ± 4.84	39.77 ± 5.14	-0.18 ± 2.82	0.714
**ICP- Whole**	**42.67 ± 3.20**	**42.73 ± 3.96**	**-0.06 ± 2.48**	**0.884**
ICP-S	43.43 ± 3.79	43.05 ± 4.35	0.38 ± 3.82	0.565
ICP-T	40.97 ± 4.24	41.24 ± 4.66	-0.27 ± 4.01	0.691
ICP-I	43.88 ± 3.46	44.25 ± 4.34	-0.37 ± 2.88	0.452
ICP-N	42.42 ± 3.80	42.40 ± 4.53	0.02 ± 2.95	0.964
**DCP- Whole**	**26.41 ± 3.98**	**27.22 ± 4.38**	**-0.81 ± 2.77**	**0.094**
DCP-S	28.05 ± 4.64	28.19 ± 4.50	-0.13 ± 3.53	0.824
DCP-T	26.28 ± 5.17	27.07 ± 5.33	-0.79 ± 3.27	0.163
DCP-I	26.51 ± 4.61	27.24 ± 4.77	-0.72 ± 3.84	0.273
DCP-N	24.81 ± 5.00	26.39 ± 5.48	-1.58 ± 3.90	*0.022*

S, superior; T, temporal; I, inferior; N, nasal. Values are presented as mean ± SD. ^†^Values were compared by paired t-test. Italic values indicate significance at P<0.05. Parameters commonly used are in bold.

For macular 6×6mm scan mode, VD in 1-3mm and 3-6mm annular areas were compared before and after mydriasis (**Supplementary Tables 3**, **4**). There was no significant difference in macular VD in the whole annular area and four quadrants of SVP (*P*=0.114-0.731), ICP (*P*=0.124-0.985), and DCP (*P*=0.057-0.974), except SVP in nasal quadrant (*P*=0.042) of 1-3mm annular area.

For optic nerve 4.5×4.5mm scan mode, VD in 2-4mm annular area was compared before and after mydriasis (**Table 3**). There was no significant difference in peripapillary VD in the whole annular area, superior, inferior, and eight sectors (*P*=0.060-0.870).

**Table 3:** Peripapillary vessel density in 2-4mm annular area of optic nerve 4.5×4.5mm scan before and after mydriasis.

Area	Before mydriasis (%)	After mydriasis (%)	Difference (%)	*P* value
**Whole**	**64.25 ± 3.63**	**64.09 ± 3.09**	**0.16 ± 2.65**	**0.717^†^**
S	65.00 ± 4.81	65.09 ± 3.91	-0.09 ± 3.00	0.861^†^
I	63.43 ± 4.18	62.99 ± 4.62	0.44 ± 3.66	0.483^†^
NS	57.67 ± 7.54	57.57 ± 7.12	0.09 ± 4.27	0.743^*^
NI	51.49 ± 7.42	51.93 ± 7.25	-0.44 ± 6.80	0.566^*^
IN	61.57 ± 6.59	60.23 ± 8.16	1.34 ± 4.08	0.060^†^
IT	76.18 ± 6.66	75.70 ± 7.22	0.49 ± 4.03	0.479^†^
TI	68.93 ± 6.68	68.51 ± 7.21	0.42 ± 5.53	0.870^*^
TS	66.70 ± 7.12	67.24 ± 6.03	-0.54 ± 4.40	0.473^†^
ST	75.82 ± 5.79	76.30 ± 6.41	-0.49 ± 3.35	0.396^†^
SN	64.66 ± 7.53	64.23 ± 7.12	0.43 ± 4.47	0.573^†^

S, superior; I, inferior; NS, nasosuperior; NI, nasoinferior; IN, inferonasal; IT, inferotemporal; TI, temporoinferior; TS, temporosuperior; ST, superotemporal; SN, superonasal. Values are presented as mean ± SD. Values were compared by ^*^Wilcoxon sign-rank tests or ^†^paired t-test. Parameters commonly used are in bold.

### 3.2 Comparison of choroidal and retinal thickness before and after mydriasis.

For macular 3×3mm scan mode, the thickness of the choroid, outer retina, whole retina, RNFL, and GCC in foveal (0-1mm) and 1-3mm annular area was compared before and after mydriasis (**Table 4**; **Supplementary Table 5**). The thickness of the choroid, outer retina, and whole retina was significantly different (*P*<0.01). RNFL (*P*=0.048) and GCC (*P*=0.013) in the temporal quadrant were significantly increased, while there was no significant difference in the other areas (*P*=0.095-0.962).

**Table 4:** Thickness of choroid, outer retina, whole retina, retinal nerve fiber layer (RNFL), and the ganglion cell complex (GCC) in macular 3×3mm scan before and after mydriasis.

Layer	Area	Before mydriasis (μm, mean ± SD)	After mydriasis (μm, mean ± SD)	Difference (μm, mean ± SD)	*P* value
Choroid	0-1mm Whole	337.16 ± 104.82	328.83 ± 101.12	8.34 ± 13.59	<0.001^*^
	1-3mm Whole	332.32 ± 101.64	325.83 ± 100.50	6.49 ± 9.08	*<0.001^†^*
Outer Retina	0-1mm Whole	186.22 ± 12.28	187.07 ± 12.49	-0.85 ± 1.09	*<0.001^†^*
	1-3mm Whole	173.51 ± 8.59	174.50 ± 8.64	-0.99 ± 0.90	*<0.001^†^*
Whole Retina	0-1mm Whole	258.13 ± 22.17	259.35 ± 22.54	-1.22 ± 1.37	*<0.001^†^*
	1-3mm Whole	329.06 ± 14.72	330.29 ± 14.74	-1.23 ± 1.11	*<0.001^†^*
RNFL	0-1mm Whole	14.18 ± 1.36	14.16 ± 1.53	0.02 ± 0.40	0.670^*^
	1-3mm Whole	25.21 ± 2.74	25.08 ± 2.84	0.12 ± 0.55	0.204^†^
GCC	0-1mm Whole	44.01 ± 10.51	44.12 ± 10.63	-0.11 ± 0.94	0.482^†^
	1-3mm Whole	112.92 ± 7.85	113.10 ± 8.04	-0.18 ± 0.72	0.161^†^

Values are presented as mean ± SD. Values were compared by ^*^Wilcoxon sign-rank tests or ^†^paired t-test. Italic values indicate significance at P<0.05.

For macular 6×6mm scan mode, the thickness of choroid, outer retina, whole retina, RNFL, and GCC in foveal (0-1mm), 1-3mm, and 3-6 mm annular area were compared before and after mydriasis (**Supplementary Table 6**). The thickness of the choroid, outer retina, and whole retina was significantly different in most sectors (all *P*<0.05), while RNFL and GCC were not (all *P*>0.05).

For optic nerve 4.5×4.5mm scan mode, the thickness of choroid, outer retina, whole retina, RNFL, and GCC in 0-2mm and 2-4mm annular area were compared before and after mydriasis (**Table 5**; **Supplementary Table 7**). The thickness of the choroid was significantly decreased (*P*<0.018). The thickness of the whole retina and GCC was significantly different in some sectors (*P*=0.001-0.049). The thickness of the outer retina and RNFL was not significantly different in most sectors (*P*=0.051-0.883).

**Table 5:** Thickness of choroid, outer retina, whole retina, retinal nerve fiber layer (RNFL), and the ganglion cell complex (GCC) in optic nerve 4.5×4.5mm scan before and after mydriasis.

Layer	Area	Before mydriasis (μm, mean ± SD)	After mydriasis (μm, mean ± SD)	Difference (μm, mean ± SD)	*P* value
**Choroid**	**0-2mm Whole**	**76.65 ± 24.60**	**74.30 ± 24.98**	**2.35 ± 3.14**	<0.001^*^
	**2-4mm Whole**	**190.47 ± 48.30**	**187.72 ± 48.40**	**2.75 ± 5.98**	0.002^*^
	2-4mm S	198.71 ± 49.61	196.04 ± 49.32	2.67 ± 8.75	0.004^*^
	2-4mm I	181.51 ± 50.56	178.65 ± 51.06	2.86 ± 4.80	<0.001^*^
**Outer retina**	**0-2mm Whole**	**45.03 ± 14.15**	**45.25 ± 14.65**	**-0.22 ± 2.62**	0.844^*^
	**2-4mm Whole**	**126.33 ± 8.91**	**126.51 ± 8.70**	**-0.18 ± 1.28**	0.164^*^
	2-4mm S	128.25 ± 11.25	128.18 ± 10.96	0.07 ± 2.99	0.555^*^
	2-4mm I	124.24 ± 9.25	124.70 ± 9.13	-0.46 ± 1.84	0.051^*^
**Whole Retina**	**0-2mm Whole**	**499.33 ± 88.73**	**500.79 ± 86.07**	**-1.45 ± 11.07**	**0.443^†^**
	**2-4mm Whole**	**321.29 ± 21.08**	**322.08 ± 20.71**	**-0.79 ± 2.65**	0.007^*^
	2-4mm S	325.08 ± 24.08	325.81 ± 24.28	-0.73 ± 5.41	0.025^*^
	2-4mm I	317.16 ± 21.80	317.97 ± 21.15	-0.81 ± 3.40	0.010^*^
**RNFL**	**0-2mm Whole**	**432.97 ± 80.85**	**434.50 ± 78.17**	**-1.52 ± 13.36**	**0.504^†^**
	**2-4mm Whole**	**130.22 ± 17.33**	**130.76 ± 17.51**	**-0.53 ± 2.95**	0.036^*^
	2-4mm S	131.08 ± 24.59	131.96 ± 25.93	-0.88 ± 8.40	0.055^*^
	2-4mm I	129.28 ± 20.33	129.38 ± 19.63	-0.11 ± 5.22	0.105^*^
**GCC**	**0-2mm Whole**	**445.24 ± 82.34**	**446.53 ± 79.65**	**-1.28 ± 12.81**	**0.557^†^**
	**2-4mm Whole**	**165.57 ± 18.33**	**166.17 ± 18.44**	**-0.60 ± 2.87**	0.006^*^
	2-4mm S	167.22 ± 24.59	168.05 ± 25.59	-0.83 ± 8.22	0.021^*^
	2-4mm I	163.76 ± 21.37	164.06 ± 20.75	-0.30 ± 4.67	0.042^*^

S, superior; I, inferior. Values are presented as mean ± SD. Values were compared by ^*^Wilcoxon sign-rank tests or ^†^paired t-test. Italic values indicate significance at P<0.05. Parameters commonly used are in bold.

### 3.3 Agreement of OCTA parameters before and after mydriasis.

For macular 3×3mm scan mode, ICCs of FAZ area and perimeter were 0.996 and 0.935 (**Supplementary Table 8**). The ICCs and 95% LoA on Bland-Altman plots are displayed in **Supplementary Table 8**.

For macular 3×3mm, 6×6mm, and 4.5×4.5mm scan modes, ICCs of VD ranged from 0.352 to 0.884 before and after mydriasis (all ICC<0.900). The ICCs and 95% LoA are displayed in **Supplementary Tables 9**–**12**.

For macular 3×3mm, 6×6mm, and 4.5×4.5mm scan modes, ICCs of choroidal and retinal thickness ranged from 0.902 to 0.998 before and after mydriasis, except thickness of the outer retina, RNFL, and GCC of 2-4mm superonasal region in 4.5×4.5mm scan mode (0.860-0.870), and RNFL thickness of some areas in 6×6mm scan mode (0.709-0.856). The ICCs and 95% LoA are displayed in **Supplementary Tables 13**–**15**.

## 4 Discussion

Mydriasis is commonly used to achieve a greater fundus view before the operation or retinal examination in clinical practice. It is vital to investigate the effects of mydriasis on retinal and choroidal metrics to distinguish these findings from pathological alterations. In this observational study, we used SS-OCTA to evaluate the effect of topical 0.5% tropicamide and 0.5% phenylephrine mixture eye drops on VD and thickness in different parts of the fundus. Nevertheless, neither the macular nor peripapillary region manifested any statistically significant difference in retinal VD and RNFL/GCC thickness with SS-OCTA in the current study, while choroidal thickness was reduced, and retinal thickness was increased in the macular region after mydriasis.

Our findings are in accordance with some previous studies on SD-OCTA. Several previous studies investigating the effects of pupil dilation on SD-OCTA metrics indicated that pharmacologic mydriasis did not influence retinal VD in the macular and peripapillary region (10, 14, 15). However, another previous study using the combination of 0.5% tropicamide and 0.5% phenylephrine (same as the present study) showed reduced the retinal VD within the peripapillary area, but not within the macular area on SD-OCTA (6). Different findings may be caused by the different equipment, scanning windows, and analyzing software. Results of the present study showing no significant changes in retinal VD after mydriasis may be also due to better visualization of retinal vessels through photopic pupils using SS-OCTA. Notably, the high-resolution, high-penetration and roll-off performance of this system provide more details of the retina and choroid at full depth (16). Moreover, this system detects the movement of red blood cells in the vascular cavity through continuous scanning and then integrates the three-dimensional image reconstruction of the fundus microvascular structure (17). Additionally, because of the longer laser wavelength, ultrahigh imaging speeds, and high-power function of eliminating motion artifacts, SS-OCTA improves visualization of structural details in the fundus even in a 2.5mm photopic pupil (18).

Besides the optics of SS-OCTA, the effects of the mydriatic agent should also be considered in the present study. Tropicamide is a vasoactive agent commonly used in clinical work to induce mydriasis, targeting and blocking cholinergic receptors (19, 20). However, evidences suggest that retinal microvasculature is not dominated by sympathetic and parasympathetic neurons directly (21, 22). Both clinical studies and animal studies indicated the nutrition and oxygenation of the retina are thought to be maintained via autoregulation (21, 23, 24). Although neuron domination does not affect the retinal microcirculation, acetylcholine (Ach) could stimulate the synthesis of nitric oxide (NO), which has a potential effect on retinal endothelial cells and smooth muscles (25). The fact that no smooth muscles existed in the macula vessels might explain why macular VD was not affected by mydriasis.

The present study indicated reduced choroidal thickness and increased retinal thickness in the macular region on SS-OCTA after mydriasis, while no statistical difference in retinal thickness in the peripapillary region. A previous study about choroidal thickness in children revealed complete mydriasis induced by the compound tropicamide led to choroidal thinning (5). The reason for the thinning choroid influenced by mydriasis is not very clear. A possible mechanism is that mydriatics could influence choroidal thickness measurement by changing the uveal structures. Because mydriatics result in ciliary muscle relaxation, and the non-vascular smooth muscle cells are connected to the ciliary muscle fibers (26), mydriatics can cause contraction of the non-vascular smooth muscle cells, leading to contraction of the choroidal vascular bed, decreased mechanical traction, fluid efflux out of the choroid, and choroidal thinning by constriction (27–31). In contrast to these results, two previous studies have reported that the choroidal thickness measurement was not influenced by mydriasis both in normal and glaucomatous eyes (32, 33). It was proposed that the effects of mydriasis on the choroid thickness are caused by the interplay of choroidal non-vascular smooth muscle, choroidal blood flow, and choroidal lymphatics, and change in the balance of the interplay may lead to different effects of mydriasis (32).

A previous study with the RTVue-100 OCT revealed that the RNFL and GCC thickness measurements were not influenced by pupil dilation either in normal subjects or in patients with glaucoma (11). Another previous study on SD-OCT suggested RNFL, GCC, ONH rim volume, and image quality score were not influenced by pupil dilation either for the glaucoma patients or for healthy subjects (34). Similarly, the present study indicated signal strength index and the thickness of RNFL and GCC were not altered by mydriasis. These results suggested that RNFL and GCC thickness measurements might not be interchangeable between mydriatic and non-mydriatic conditions. For patients with open-angle glaucoma, this may be an issue that needs to be paid more attention in clinical practice. However, for patients with close-angle glaucoma, this may not be that important since mydriasis is usually contraindicated in these patients.

Due to the wide scanning mode of SS-OCTA, the blood vessels and structures of the peripheral retina can be detected without mydriasis, which is particularly beneficial to diabetic patients. In diabetes, sustained hyperglycemia leads to thinned iridis, weakened dilator pupillae muscle, and degeneration of the nerve controlling the ciliary muscle (35). Therefore, some diabetic patients may need much longer time to fully dilate the pupil (9). But using nonmydriatic SS-OCTA, retinal vessel image acquisition is possible without the need to the dilate pupil, if fundus VDs are the key parameters to observe. Nonmydriatic SS-OCTA examination is also applicable in patients with narrow anterior chamber angle, who need more caution in mydriasis, to prevent possible acute angle closure.

Of note, we further investigated the agreement of OCTA parameters before and after mydriasis and calculated the 95% LoA. The results are clinically important for the interchange and interpretation of OCTA parameters under different conditions (ie. non-mydriasis and mydriasis). If a patient had OCTA examination under mydriasis at baseline and had the examination under non-mydriasis condition in the next follow-up, it would be difficult to compare the results between the two sections. However, with the information we provided in the present study, it is possible the estimate the follow-up OCTA parameters that should have been measured under mydriasis, using the OCTA parameters obtained under non-mydriasis condition, although the accuracy of such estimations need to be validated in further studies.

The highlight of this study lies in the application of the SS-OCTA system. Our findings can also provide implications for clinical application. The present study was limited by the small view of SS-OCTA covering a retinal area of 6×6 mm^2^. How the SS-OCTA metrics under a larger field of view are affected by mydriasis needs to be further investigated. Additionally, healthy subjects were involved in this study. Patients with glaucoma or retinal disorders may have less satisfactory fixation during OCTA examinations, and mydriasis may aggravate the situation. Thus, their OCTA metrics may be altered after mydriasis, especially the VD metrics. In a further study, the effects of mydriasis in these patients should be explored to improve our knowledge in this field. Another limitation lies on the missing analysis of test-retest variation for the OCTA examination. However, in the present study a single operator and the same time point of examination maybe helpful to minimize such variation.

In conclusion, mydriasis did not affect signal strength, FAZ parameters, and retinal VD in most macula and peripapillary regions. Mydriasis reduced the thickness of the choroid, while increased the thickness of retinal layers in some sectors. Therefore, SS-OCTA measurement of retinal VD in healthy subjects can be captured with a dilated or undilated pupil, which largely remains unaffected by mydriasis.

## Data availability statement

The original contributions presented in the study are included in the article/**Supplementary Material**. Further inquiries can be directed to the corresponding authors.

## Ethics statement

The studies involving humans were approved by Research Ethics Committee of Guangdong General Hospital. The studies were conducted in accordance with the local legislation and institutional requirements. Written informed consent for participation in this study was provided by the participants’ legal guardians/next of kin.

## Author contributions

FZ: Conceptualization, Data curation, Formal analysis, Methodology, Resources, Visualization, Writing – original draft, Writing – review & editing. Y’aL: Data curation, Formal analysis, Methodology, Software, Writing – original draft. ZD: Data curation, Formal analysis, Software, Writing – original draft. HS: Data curation, Formal analysis, Writing – original draft. LX: Methodology, Writing – original draft. YYL: Formal analysis, Writing – original draft. SZ: Data curation, Writing – review & editing. WS: Supervision, Writing – review & editing. HY: Funding acquisition, Supervision, Writing – review & editing. YH: Conceptualization, Funding acquisition, Methodology, Project administration, Resources, Supervision, Validation, Visualization, Writing – review & editing.
