# Supplementary material for: Effect of mydriasis on macular and peripapillary metrics in swept-source optical coherence tomography angiography
Source: Front Endocrinol (Lausanne). 2024 Feb 28;15:1292255. doi: 10.3389/fendo.2024.1292255 (PMC10933103; doi:10.3389/fendo.2024.1292255)
Supplement: Supplementary file 1 [file Table_1.docx]

Supplementary Table 1. Characteristics of study population.

| Characteristics | Study subjects |
| --- | --- |
| Number of eyes, patients | 35 |
| Age, year | 26(23, 34) |
| Gender, male/female | 16/19(45.7%/54.3%) |
| Best corrected visual acuity, LogMAR | 0.00(0.00, 0.00) |
| Spherical equivalent, diopter | -1.13(-1.50, -0.88) |
| Intraocular pressure, mmHg | 15.16 ± 2.32 |

LogMAR, logarithm of the minimum angle of resolution. Values are presented as mean ± SD or median (25th and 75th quartiles).

Supplementary Table 2. Signal strength of the three scans before and after mydriasis.

| Scan | Before mydriasis | After mydriasis | *P* value^*^ |
| --- | --- | --- | --- |
| Macular 3×3 mm | 10 (10,10) | 10 (9,10) | 0.477 |
| Macular 6×6 mm | 10 (9,10) | 10 (9,10) | 0.806 |
| Optic nerve 4.5×4.5 mm | 9 (9,10) | 10 (9,10) | 0.275 |

Values are presented as median (25th and 75th quartiles). ^*^Values were compared by Wilcoxon sign-rank tests.

Supplementary Table 3. Macular vessel density of superficial vascular plexus (SVP), intermediate capillary plexus (ICP), and deep capillary plexus (DCP) in 1-3mm annular area of macular 6×6mm scan before and after mydriasis.

| Area | Before mydriasis (%) | After mydriasis (%) | Difference (%) | *P* value |
| --- | --- | --- | --- | --- |
| **1-3mm SVP- Whole** | **43.10±4.36** | **43.67±5.13** | **-0.57±2.96** | **0.260^†^** |
| 1-3mm SVP-S | 48.18±4.93 | 47.80±6.16 | 0.38±5.33 | 0.623^*^ |
| 1-3mm SVP-T | 36.75±5.11 | 37.53±5.38 | -0.78±2.97 | 0.128^†^ |
| 1-3mm SVP-I | 44.86±5.42 | 46.05±6.45 | -1.19±4.35 | 0.114^†^ |
| 1-3mm SVP-N | 42.67±4.94 | 43.37±5.60 | -0.70±3.47 | *0.042** |
| **1-3mm ICP- Whole** | **37.72±4.29** | **38.35±4.94** | **-0.63±3.62** | **0.310^†^** |
| 1-3mm ICP-S | 36.57±5.25 | 37.42±7.84 | -0.86±7.60 | 0.201^*^ |
| 1-3mm ICP-T | 37.42±5.62 | 38.56±5.04 | -1.14±4.62 | 0.207^*^ |
| 1-3mm ICP-I | 37.99±5.06 | 38.62±5.21 | -0.63±5.32 | 0.489^†^ |
| 1-3mm ICP-N | 38.88±5.17 | 38.78±5.21 | 0.11±4.55 | 0.544^*^ |
| **1-3mm DCP- Whole** | **11.06±3.83** | **12.17±4.68** | **-1.11±3.62** | **0.079^†^** |
| 1-3mm DCP-S | 11.07±4.49 | 12.11±5.43 | -1.04±5.49 | 0.270^†^ |
| 1-3mm DCP-T | 13.06±5.80 | 13.98±6.59 | -0.92±4.92 | 0.441^*^ |
| 1-3mm DCP-I | 10.55±5.42 | 11.34±4.78 | -0.79±4.76 | 0.331^†^ |
| 1-3mm DCP-N | 9.56±4.24 | 11.23±5.03 | -1.67±5.03 | 0.057^†^ |

S, superior; T, temporal; I, inferior; N, nasal. Values are presented as mean ± SD. Values were compared by ^*^Wilcoxon sign-rank tests or ^†^paired *t*-test. Italic values indicate significance at *P*<0.05. Parameters commonly used are in bold.

Supplementary Table 4. Macular vessel density of superficial vascular plexus (SVP), intermediate capillary plexus (ICP), and deep capillary plexus (DCP) in 3-6mm annular area of macular 6×6mm scan before and after mydriasis.

| Area | Before mydriasis (%) | After mydriasis (%) | Difference (%) | *P* value |
| --- | --- | --- | --- | --- |
| **3-6mm SVP-Whole** | **37.33±3.89** | **37.65±4.06** | **-0.33±2.02** | **0.346^†^** |
| 3-6mm SVP-S | 38.04±4.64 | 37.79±5.02 | 0.25±3.32 | 0.731^*^ |
| 3-6mm SVP-T | 28.97±4.74 | 29.30±4.72 | -0.33±2.52 | 0.443^†^ |
| 3-6mm SVP-I | 37.47±4.26 | 38.26±5.49 | -0.79±3.12 | 0.143^†^ |
| 3-6mm SVP-N | 44.84±5.08 | 45.27±4.70 | -0.43±3.71 | 0.494^†^ |
| **3-6mm ICP- Whole** | **35.05±3.65** | **35.04±3.73** | **0.01±3.01** | **0.985^†^** |
| 3-6mm ICP-S | 33.94±4.36 | 33.30±6.10 | 0.64±4.47 | 0.922^†^ |
| 3-6mm ICP-T | 36.10±4.56 | 36.02±4.58 | 0.08±4.52 | 0.916^†^ |
| 3-6mm ICP-I | 32.99±5.84 | 32.89±5.39 | 0.10±7.30 | 0.756^*^ |
| 3-6mm ICP-N | 37.14±4.95 | 37.92±5.27 | -0.78±4.52 | 0.124^*^ |
| **3-6mm DCP- Whole** | **21.17±4.62** | **21.17±4.76** | **0.00±3.75** | **0.974^*^** |
| 3-6mm DCP-S | 20.00±5.15 | 19.76±5.68 | 0.24±5.35 | 0.589^*^ |
| 3-6mm DCP-T | 27.81±6.98 | 27.38±7.02 | 0.44±6.82 | 0.707^†^ |
| 3-6mm DCP-I | 18.98±6.79 | 18.38±6.08 | 0.60±7.15 | 0.624^†^ |
| 3-6mm DCP-N | 17.86±6.83 | 19.13±6.37 | -1.26±6.13 | 0.231^†^ |

S, superior; T, temporal; I, inferior; N, nasal. Values are presented as mean ± SD. Values were compared by ^*^Wilcoxon sign-rank tests or ^†^paired *t*-test. Parameters commonly used are in bold.

Supplementary table 5. Thickness of choroid, outer retina, whole retina, retinal nerve fiber layer (RNFL), and the ganglion cell complex (GCC) in macular 3×3mm scan before and after mydriasis.

| Layer | Area | Before mydriasis  (μm, mean±SD) | After mydriasis (μm, mean±SD) | Difference  (μm, mean±SD) | *P* value |
| --- | --- | --- | --- | --- | --- |
| Choroid | 1-3mm S | 341.88±108.63 | 334.78±104.92 | 7.10±10.38 | *<0.001^†^* |
|  | 1-3mm T | 348.84±102.18 | 341.26±100.85 | 7.58±11.29 | *<0.001^*^* |
|  | 1-3mm I | 327.84±99.43 | 322.64±100.35 | 5.21±11.33 | *0.010^†^* |
|  | 1-3mm N | 310.72±105.89 | 304.66±104.38 | 6.06±9.57 | *0.001^†^* |
| Outer Retina | 1-3mm S | 171.62±8.28 | 172.23±8.22 | -0.61±1.15 | *0.003^†^* |
|  | 1-3mm T | 175.95±9.71 | 176.77±9.96 | -0.81±1.37 | *0.001^†^* |
|  | 1-3mm I | 170.89±9.16 | 172.26±9.37 | -1.37±1.29 | *<0.001^†^* |
|  | 1-3mm N | 175.54±8.80 | 176.71±8.69 | -1.17±1.46 | *<0.001^†^* |
| Whole Retina | 1-3mm S | 335.53±14.40 | 336.56±14.43 | -1.04±1.58 | *<0.001^†^* |
|  | 1-3mm T | 319.20±15.40 | 320.63±15.57 | -1.43±1.36 | *<0.001^†^* |
|  | 1-3mm I | 327.73±15.70 | 329.09±15.81 | 1.37±1.29 | *<0.001^†^* |
|  | 1-3mm N | 333.81±15.21 | 334.91±14.99 | 1.10±1.15 | *<0.001^†^* |
| RNFL | 1-3mm S | 31.33±5.02 | 31.12±5.09 | 0.21±0.94 | 0.197^†^ |
|  | 1-3mm T | 18.54±1.35 | 18.67±1.55 | -0.13±0.39 | *0.048^†^* |
|  | 1-3mm I | 26.97±4.00 | 26.73±3.89 | 0.24±0.84 | 0.095^†^ |
|  | 1-3mm N | 24.02±3.19 | 23.85±3.29 | 0.17±0.83 | 0.231^†^ |
| GCC | 1-3mm S | 120.57±8.72 | 120.80±8.86 | -0.23±1.15 | 0.253^†^ |
|  | 1-3mm T | 102.11±8.11 | 102.45±8.13 | -0.34±0.77 | *0.013^†^* |
|  | 1-3mm I | 114.39±9.26 | 114.52±9.55 | -0.13±1.10 | 0.499^†^ |
|  | 1-3mm N | 114.67±8.85 | 114.68±9.00 | -0.01±1.07 | 0.962^†^ |

S, superior; T, temporal; I, inferior; N, nasal. Values are presented as mean ± SD. Values were compared by ^*^Wilcoxon sign-rank tests or ^†^paired *t*-test. Italic values indicate significance at *P*<0.05.

Supplementary table 6. Thickness of choroid, outer retina, whole retina, retinal nerve fiber layer (RNFL), and the ganglion cell complex (GCC) in macular 6×6mm scan before and after mydriasis.

| Layer | Area | Before mydriasis  (μm, mean±SD) | After mydriasis  (μm, mean±SD) | Difference  (μm, mean±SD) | *P* value |
| --- | --- | --- | --- | --- | --- |
| **Choroid** | **0-1mm Whole** | **327.56±101.07** | **322.29±99.96** | **5.27±17.03** | ***0.006^*^*** |
|  | **1-3mm Whole** | **325.12±99.60** | **321.04±99.54** | **4.07±12.29** | **0.058^†^** |
|  | 1-3mm S | 334.77±102.17 | 331.38±102.78 | 3.40±15.89 | *0.006^*^* |
|  | 1-3mm T | 340.71±98.34 | 337.06±98.83 | 3.64±15.40 | *0.018^*^* |
|  | 1-3mm I | 325.81±101.17 | 322.86±102.97 | 2.95±16.72 | *0.004^*^* |
|  | 1-3mm N | 299.23±104.38 | 292.87±102.27 | 6.36±12.15 | *0.001^*^* |
|  | **3-6mm Whole** | **308.67±88.05** | **306.73±93.35** | **1.93±12.65** | ***0.011^*^*** |
|  | 3-6mm S | 332.31±82.13 | 332.72±94.86 | -0.41±25.22 | *0.027^*^* |
|  | 3-6mm T | 341.46±101.52 | 337.74±100.77 | 3.72±10.61 | *0.002^*^* |
|  | 3-6mm I | 317.22±92.26 | 314.16±94.78 | 3.07±11.31 | *0.003^*^* |
|  | 3-6mm N | 243.78±95.07 | 242.46±101.47 | 1.32±20.90 | *0.003^*^* |
| **Outer Retina** | **0-1mm Whole** | **186.78±13.87** | **187.75±13.58** | **-0.97±1.38** | ***<0.001^*^*** |
|  | **1-3mm Whole** | **172.76±9.13** | **173.50±9.30** | **-0.74±0.81** | ***<0.001^†^*** |
|  | 1-3mm S | 172.25±8.63 | 172.67±9.06 | -0.42±1.57 | 0.179^*^ |
|  | 1-3mm T | 174.11±9.57 | 174.61±10.27 | -0.51±1.73 | *0.026^*^* |
|  | 1-3mm I | 168.20±9.40 | 169.38±9.43 | -1.18±1.29 | *<0.001^†^* |
|  | 1-3mm N | 176.50±9.74 | 177.33±9.53 | -0.83±1.51 | *0.010^*^* |
|  | **3-6mm Whole** | **148.14±7.73** | **148.82±8.00** | **-0.68±0.80** | ***<0.001^†^*** |
|  | 3-6mm S | 151.60±7.54 | 151.95±7.67 | -0.36±0.97 | *0.037^†^* |
|  | 3-6mm T | 150.70±8.13 | 151.34±9.28 | -0.64±2.51 | 0.142^†^ |
|  | 3-6mm I | 141.04±7.88 | 142.07±8.07 | -1.03±1.14 | *<0.001^†^* |
|  | 3-6mm N | 149.08±9.17 | 149.77±9.23 | -0.69±1.85 | *0.035^†^* |
| **Whole Retina** | **0-1mm Whole** | **252.33±21.68** | **253.19±21.49** | **-0.87±1.39** | ***0.001^†^*** |
|  | **1-3mm Whole** | **328.93±15.08** | **329.94±15.17** | **-1.01±0.86** | ***<0.001^†^*** |
|  | 1-3mm S | 333.87±14.89 | 334.65±14.88 | -0.77±1.14 | *<0.001^†^* |
|  | 1-3mm T | 318.97±15.52 | 320.15±16.17 | -1.18±1.71 | *<0.001^*^* |
|  | 1-3mm I | 329.32±14.90 | 330.39±15.14 | -1.07±1.28 | *<0.001^†^* |
|  | 1-3mm N | 333.56±15.97 | 334.56±15.62 | -1.00±1.06 | *<0.001^†^* |
|  | **3-6mm Whole** | **286.78±13.39** | **287.73±13.68** | **-0.95±0.94** | ***<0.001^†^*** |
|  | 3-6mm S | 289.71±13.43 | 290.46±13.59 | -0.75±1.24 | *0.001^†^* |
|  | 3-6mm T | 272.57±13.53 | 273.71±15.04 | -1.14±2.65 | *0.001^*^* |
|  | 3-6mm I | 276.84±14.36 | 277.82±14.55 | -0.98±1.50 | *<0.001^†^* |
|  | 3-6mm N | 307.73±15.31 | 308.7±15.24 | -0.97±2.48 | *0.031^*^* |
| **RNFL** | **0-1mm Whole** | **14.14±1.30** | **14.36±1.03** | **-0.23±0.90** | **0.287^*^** |
|  | **1-3mm Whole** | **25.25±2.35** | **25.28±2.08** | **-0.03±0.62** | **0.694^*^** |
|  | 1-3mm S | 29.31±3.44 | 29.28±3.30 | 0.03±0.96 | 0.864^†^ |
|  | 1-3mm T | 18.35±1.46 | 18.61±1.20 | -0.26±0.72 | *0.019^*^* |
|  | 1-3mm I | 29.27±2.98 | 29.31±2.68 | -0.04±0.97 | 0.827^†^ |
|  | 1-3mm N | 24.08±2.40 | 23.94±2.20 | 0.13±0.73 | 0.294^†^ |
|  | **3-6mm Whole** | **46.12±6.49** | **46.20±6.36** | **-0.08±0.95** | **0.883^*^** |
|  | 3-6mm S | 49.12±7.22 | 49.03±7.17 | 0.09±1.03 | 0.607^†^ |
|  | 3-6mm T | 24.29±3.11 | 24.72±2.86 | -0.43±1.78 | 0.162^†^ |
|  | 3-6mm I | 51.11±8.99 | 51.25±8.56 | -0.14±1.32 | 0.768**^*^** |
|  | 3-6mm N | 59.93±9.81 | 59.80±10.00 | 0.13±1.70 | 0.658^†^ |
| **GCC** | **0-1mm Whole** | **38.32±8.10** | **38.22±8.11** | **0.10±0.65** | **0.368^†^** |
|  | **1-3mm Whole** | **112.69±7.75** | **112.91±7.52** | **-0.23±0.52** | ***0.014^†^*** |
|  | 1-3mm S | 117.26±8.59 | 117.34±8.27 | -0.08±1.07 | 0.679^†^ |
|  | 1-3mm T | 102.92±7.79 | 103.40±7.55 | -0.48±0.86 | *0.002^†^* |
|  | 1-3mm I | 117.12±7.75 | 117.32±7.60 | -0.20±1.10 | 0.298^†^ |
|  | 1-3mm N | 113.43±7.72 | 113.60±7.61 | -0.16±0.76 | 0.211^†^ |
|  | **3-6mm Whole** | **101.04±8.50** | **101.14±8.44** | **-0.10±0.77** | **0.461^†^** |
|  | 3-6mm S | 101.62±8.99 | 101.69±9.13 | -0.07±1.01 | 0.685^†^ |
|  | 3-6mm T | 82.52±7.08 | 82.74±7.02 | -0.22±1.21 | 0.301^†^ |
|  | 3-6mm I | 100.51±9.98 | 100.45±9.87 | 0.05±1.31 | 0.682**^*^** |
|  | 3-6mm N | 119.41±10.67 | 119.58±10.70 | -0.17±1.50 | 0.499^†^ |

S, superior; T, temporal; I, inferior; N, nasal. Values are presented as mean ± SD. Values were compared by^*^Wilcoxon sign-rank tests or ^†^paired *t*-test. Italic values indicate significance at *P*<0.05. Parameters commonly used are in bold.

Supplementary table 7. Thickness of choroid, outer retina, whole retina, retinal nerve fiber layer (RNFL), and the ganglion cell complex (GCC) in optic nerve 4.5×4.5mm scan before and after mydriasis.

| Layer | Area | Before mydriasis  (μm, mean±SD) | After mydriasis  (μm, mean±SD) | Difference  (μm, mean±SD) | *P* value |
| --- | --- | --- | --- | --- | --- |
| Choroid | 2-4mm NS | 217.58±50.01 | 215.02±51.24 | 2.56±9.04 | *0.005^*^* |
|  | 2-4mm NI | 212.92±50.72 | 210.29±51.21 | 2.63±5.89 | *0.012^†^* |
|  | 2-4mm IN | 176.72±51.46 | 174.06±51.88 | 2.66±5.93 | *0.004^*^* |
|  | 2-4mm IT | 161.89±52.01 | 158.76±52.81 | 3.13±5.93 | *0.004^†^* |
|  | 2-4mm TI | 165.53±61.15 | 162.34±60.52 | 3.20±5.01 | *0.001^†^* |
|  | 2-4mm TS | 175.63±56.57 | 173.21±55.73 | 2.43±6.84 | *0.013^*^* |
|  | 2-4mm ST | 188.79±54.56 | 185.17±53.38 | 3.62±9.56 | *0.018^*^* |
|  | 2-4mm SN | 203.42±54.31 | 201.16±52.91 | 2.26±13.89 | *0.003^*^* |
| Outer Retina | 2-4mm NS | 137.19±7.02 | 137.60±6.72 | -0.41±1.26 | 0.064^†^ |
|  | 2-4mm NI | 138.25±7.62 | 138.40±7.50 | -0.16±1.38 | 0.503^†^ |
|  | 2-4mm IN | 127.14±8.89 | 127.97±9.00 | -0.83±1.46 | *0.002^†^* |
|  | 2-4mm IT | 110.21±13.74 | 110.87±14.14 | -0.67±3.51 | 0.072^*^ |
|  | 2-4mm TI | 116.04±19.27 | 116.22±18.59 | -0.18±4.46 | 0.534^*^ |
|  | 2-4mm TS | 119.47±18.51 | 118.23±19.04 | 1.24±4.43 | 0.120^*^ |
|  | 2-4mm ST | 118.88±18.43 | 118.15±18.87 | 0.74±7.04 | 0.647^*^ |
|  | 2-4mm SN | 132.18±14.73 | 133.17±13.12 | -0.99±7.10 | 0.731^*^ |
| Whole Retina | 2-4mm NS | 299.23±29.56 | 298.93±28.85 | 0.30±6.49 | 0.359^*^ |
|  | 2-4mm NI | 280.03±27.08 | 279.69±26.60 | 0.34±4.11 | 0.578^*^ |
|  | 2-4mm IN | 324.69±36.90 | 325.19±35.41 | -0.50±7.29 | *0.047^*^* |
|  | 2-4mm IT | 368.34±36.25 | 369.52±34.71 | -1.19±10.25 | *0.005^*^* |
|  | 2-4mm TI | 306.55±25.45 | 308.99±26.87 | -2.44±7.34 | *0.001^*^* |
|  | 2-4mm TS | 306.57±22.98 | 306.22±22.39 | 0.35±6.85 | 0.085^*^ |
|  | 2-4mm ST | 370.26±34.81 | 373.89±35.99 | -3.64±12.88 | 0.051^*^ |
|  | 2-4mm SN | 342.57±44.12 | 343.48±43.83 | -0.91±19.46 | *0.016^*^* |
| RNFL | 2-4mm NS | 94.39±26.34 | 94.29±26.98 | 0.11±5.62 | 0.225^*^ |
|  | 2-4mm NI | 74.33±23.09 | 74.17±22.93 | 0.16±3.63 | 0.432^*^ |
|  | 2-4mm IN | 135.36±37.93 | 134.36±37.02 | 0.99±7.59 | 0.883^*^ |
|  | 2-4mm IT | 200.93±44.43 | 201.00±43.54 | -0.06±14.45 | 0.088^*^ |
|  | 2-4mm TI | 123.84±32.05 | 125.76±32.39 | -1.93±4.85 | *0.020^*^* |
|  | 2-4mm TS | 119.12±28.91 | 120.34±28.77 | -1.23±5.73 | 0.359^*^ |
|  | 2-4mm ST | 189.79±46.00 | 193.86±51.12 | -4.06±20.97 | 0.461^*^ |
|  | 2-4mm SN | 146.07±55.18 | 145.41±52.58 | 0.66±28.52 | 0.136^*^ |
| GCC | 2-4mm NS | 132.45±28.03 | 131.73±28.15 | 0.72±6.00 | 0.831^*^ |
|  | 2-4mm NI | 111.48±24.33 | 111.06±24.29 | 0.42±3.43 | 0.471^*^ |
|  | 2-4mm IN | 168.73±39.35 | 168.31±38.16 | 0.42±7.31 | 0.461^*^ |
|  | 2-4mm IT | 231.44±42.80 | 231.86±41.83 | -0.41±13.90 | *0.018^*^* |
|  | 2-4mm TI | 160.05±27.98 | 162.16±28.40 | -2.11±5.05 | *0.007^*^* |
|  | 2-4mm TS | 156.35±25.11 | 157.44±24.35 | -1.09±5.02 | *0.049^*^* |
|  | 2-4mm ST | 223.07±43.46 | 227.65±48.34 | -4.58±19.66 | 0.136^*^ |
|  | 2-4mm SN | 180.81±54.10 | 180.51±51.86 | 0.30±27.32 | *0.026^*^* |

NS, nasosuperior; NI, nasoinferior; IN, inferonasal; IT, inferotemporal; TI, temporoinferior; TS, temporosuperior; ST, superotemporal; SN, superonasal. Values are presented as mean ± SD. Values were compared by ^*^Wilcoxon sign-rank tests or ^†^paired *t*-test. Italic values indicate significance at *P*<0.05.

Supplemental table 8. Agreement analysis of FAZ parameters in 3×3mm scan before and after instillation of tropicamide/phenylephrine mixture eye drops.

| Parameters | *ICC* | 95% Limits of Agreement | |
| --- | --- | --- | --- |
|  |  | From | To |
| FAZ area (mm^2^) | *0.996* | -0.0266 | 0.0226 |
| Perimeter (mm) | *0.935* | -0.3675 | 0.2658 |
| CI | 0.563 | -0.1326 | 0.1943 |
| FD-300 (%) | 0.745 | -5.6940 | 4.6260 |

FAZ, Foveal avascular zone; CI, Circularity index; FD-300, Foveal vessel density in 300μm area; ICC, Intraclass correlation coefficient. Values were analyzed by ICC and Bland-Altman approach. Italic values indicate significance at *P*<0.05 and ICC>0.900.

Supplemental table 9. Agreement analysis of macular vessel density of superficial vascular plexus (SVP), intermediate capillary plexus (ICP), and deep capillary plexus (DCP) in 1-3mm annular area of 3×3mm scan before and after instillation of tropicamide/phenylephrine mixture eye drops.

| Area | *ICC* | 95% Limits of Agreement | |
| --- | --- | --- | --- |
|  |  | From | To |
| **SVP- Whole** | **0.855** | **-4.447** | **3.655** |
| SVP-S | 0.884 | -4.339 | 4.492 |
| SVP-T | 0.688 | -6.951 | 5.875 |
| SVP-I | 0.854 | -5.580 | 3.685 |
| SVP-N | 0.840 | -5.704 | 5.350 |
| **ICP- Whole** | **0.764** | **-4.914** | **4.791** |
| ICP-S | 0.562 | -7.111 | 7.859 |
| ICP-T | 0.595 | -8.133 | 7.588 |
| ICP-I | 0.731 | -6.013 | 5.270 |
| ICP-N | 0.751 | -5.763 | 5.806 |
| **DCP- Whole** | **0.782** | **-6.226** | **4.614** |
| DCP-S | 0.702 | -7.051 | 6.784 |
| DCP-T | 0.806 | -7.190 | 5.615 |
| DCP-I | 0.666 | -8.243 | 6.798 |
| DCP-N | 0.724 | -9.216 | 6.060 |

S, superior; T, temporal; I, inferior; N, nasal; ICC, Intraclass correlation coefficient. Values were analyzed by ICC and Bland-Altman approach. Parameters commonly used are in bold.

Supplemental table 10. Agreement analysis of macular vessel density of superficial vascular plexus (SVP), intermediate capillary plexus (ICP), and deep capillary plexus (DCP) in 1-3mm annular area of 6×6mm scan before and after instillation of tropicamide/phenylephrine mixture eye drops.

| Area | *ICC* | 95% Limits of Agreement | |
| --- | --- | --- | --- |
|  |  | From | To |
| **1-3mm SVP- Whole** | **0.807** | **-6.364** | **5.219** |
| 1-3mm SVP-S | 0.544 | -10.060 | 10.830 |
| 1-3mm SVP-T | 0.840 | -6.597 | 5.029 |
| 1-3mm SVP-I | 0.733 | -9.720 | 7.336 |
| 1-3mm SVP-N | 0.784 | -7.499 | 6.107 |
| **1-3mm ICP- Whole** | **0.694** | **-7.720** | **6.460** |
| 1-3mm ICP-S | 0.352 | -15.750 | 14.030 |
| 1-3mm ICP-T | 0.625 | -10.190 | 7.913 |
| 1-3mm ICP-I | 0.464 | -11.050 | 9.795 |
| 1-3mm ICP-N | 0.615 | -8.820 | 9.030 |
| **1-3mm DCP- Whole** | **0.642** | **-8.205** | **5.991** |
| 1-3mm DCP-S | 0.391 | -11.810 | 9.731 |
| 1-3mm DCP-T | 0.686 | -10.560 | 8.721 |
| 1-3mm DCP-I | 0.567 | -10.120 | 8.533 |
| 1-3mm DCP-N | 0.414 | -11.530 | 8.188 |

S, superior; T, temporal; I, inferior; N, nasal; ICC, Intraclass correlation coefficient. Values were analyzed by ICC and Bland-Altman approach. Parameters commonly used are in bold.

Supplemental table 11. Agreement analysis of macular vessel density of superficial vascular plexus (SVP), intermediate capillary plexus (ICP), and deep capillary plexus (DCP) in 3-6mm annular area of 6×6mm scan before and after instillation of tropicamide/phenylephrine mixture eye drops.

| Area | *ICC* | 95% Limits of Agreement | |
| --- | --- | --- | --- |
|  |  | From | To |
| **3-6mm SVP-Whole** | **0.872** | **-4.278** | **3.628** |
| 3-6mm SVP-S | 0.764 | -6.255 | 6.762 |
| 3-6mm SVP-T | 0.858 | -5.272 | 4.609 |
| 3-6mm SVP-I | 0.798 | -6.909 | 5.327 |
| 3-6mm SVP-N | 0.713 | -7.705 | 6.837 |
| **3-6mm ICP- Whole** | **0.667** | **-5.894** | **5.914** |
| 3-6mm ICP-S | 0.645 | -8.119 | 9.400 |
| 3-6mm ICP-T | 0.510 | -8.784 | 8.948 |
| 3-6mm ICP-I | 0.156 | -14.210 | 14.410 |
| 3-6mm ICP-N | 0.609 | -9.637 | 8.076 |
| **3-6mm DCP- Whole** | **0.680** | **-7.344** | **7.351** |
| 3-6mm DCP-S | 0.513 | -10.240 | 10.730 |
| 3-6mm DCP-T | 0.525 | -12.930 | 13.800 |
| 3-6mm DCP-I | 0.385 | -13.420 | 14.610 |
| 3-6mm DCP-N | 0.570 | -13.280 | 10.750 |

S, superior; T, temporal; I, inferior; N, nasal; ICC, Intraclass correlation coefficient. Values were analyzed by ICC and Bland-Altman approach. Parameters commonly used are in bold.

Supplemental table 12. Agreement analysis of peripapillary vessel density in 2-4mm annular area of 4.5×4.5mm scan before and after instillation of tropicamide/phenylephrine mixture eye drops.

| Area | *ICC* | 95% Limits of Agreement | |
| --- | --- | --- | --- |
|  |  | From | To |
| **Whole (%)** | **0.691** | **-5.028** | **5.356** |
| S (%) | 0.766 | -5.964 | 5.788 |
| I (%) | 0.655 | -6.735 | 7.610 |
| NS (%) | 0.830 | -8.283 | 8.471 |
| NI (%) | 0.570 | -13.770 | 12.890 |
| IN (%) | 0.849 | -6.659 | 9.341 |
| IT (%) | 0.832 | -7.403 | 8.378 |
| TI (%) | 0.683 | -10.410 | 11.260 |
| TS (%) | 0.777 | -9.169 | 8.089 |
| ST (%) | 0.849 | -7.056 | 6.082 |
| SN (%) | 0.814 | -8.336 | 9.197 |

S, superior; I, inferior; NS, nasosuperior; NI, nasoinferior; IN, inferonasal; IT, inferotemporal; TI, temporoinferior; TS, temporosuperior; ST, superotemporal; SN, superonasal; ICC, Intraclass correlation coefficient. Values were analyzed by ICC and Bland-Altman approach. Parameters commonly used are in bold.

Supplemental table 13. Agreement analysis of thickness of choroid, outer retina, whole retina, retinal nerve fiber layer (RNFL), and the ganglion cell complex (GCC) in 3×3mm scan before and after instillation of tropicamide/phenylephrine mixture eye drops.

| Layer | Location | *ICC* | 95% Limits of Agreement | |
| --- | --- | --- | --- | --- |
|  |  |  | From | To |
| **Choroid** | **0-1mm Whole** | ***0.991*** | **-18.290** | **34.970** |
|  | **1-3mm Whole** | ***0.996*** | **-11.320** | **24.290** |
|  | 1-3mm S | *0.995* | -13.240 | 27.440 |
|  | 1-3mm T | *0.988* | -14.550 | 29.710 |
|  | 1-3mm I | *0.994* | -17.000 | 27.410 |
|  | 1-3mm N | *0.996* | -12.700 | 24.830 |
| **Outer Retina** | **0-1mm Whole** | ***0.996*** | **-2.988** | **1.283** |
|  | **1-3mm Whole** | ***0.995*** | **-2.756** | **0.772** |
|  | 1-3mm S | *0.990* | -2.852 | 1.635 |
|  | 1-3mm T | *0.990* | -3.505 | 1.880 |
|  | 1-3mm I | *0.990* | -3.913 | 1.161 |
|  | 1-3mm N | *0.986* | -4.039 | 1.697 |
| **Whole Retina** | **0-1mm Whole** | ***0.998*** | **-3.904** | **1.459** |
|  | **1-3mm Whole** | ***0.997*** | **-3.412** | **0.947** |
|  | 1-3mm S | *0.994* | -4.143 | 2.069 |
|  | 1-3mm T | *0.996* | -4.095 | 1.243 |
|  | 1-3mm I | *0.997* | -3.901 | 1.162 |
|  | 1-3mm N | *0.997* | -3.345 | 1.147 |
| **RNFL** | **0-1mm Whole** | ***0.961*** | **-0.768** | **0.810** |
|  | **1-3mm Whole** | ***0.980*** | **-0.966** | **1.211** |
|  | 1-3mm S | *0.983* | -1.631 | 2.051 |
|  | 1-3mm T | *0.965* | -0.895 | 0.625 |
|  | 1-3mm I | *0.978* | -1.394 | 1.882 |
|  | 1-3mm N | *0.968* | -1.450 | 1.789 |
| **GCC** | **0-1mm Whole** | ***0.996*** | **-1.953** | **1.729** |
|  | **1-3mm Whole** | ***0.996*** | **-1.594** | **1.242** |
|  | 1-3mm S | *0.991* | -2.471 | 2.022 |
|  | 1-3mm T | *0.996* | -1.840 | 1.159 |
|  | 1-3mm I | *0.993* | -2.286 | 2.029 |
|  | 1-3mm N | *0.993* | -2.102 | 2.085 |

S, superior; T, temporal; I, inferior; N, nasal; ICC, Intraclass correlation coefficient. Values were analyzed by ICC and Bland-Altman approach. Italic values indicate significance at *P*<0.05 and ICC>0.900. Parameters commonly used are in bold.

Supplemental table 14. Agreement analysis of thickness of choroid, outer retina, whole retina, retinal nerve fiber layer (RNFL), and the ganglion cell complex (GCC) in 6×6mm scan before and after instillation of tropicamide/phenylephrine mixture eye drops.

| Layer | Location | *ICC* | 95% Limits of Agreement | |
| --- | --- | --- | --- | --- |
|  |  |  | From | To |
| **Choroid** | **0-1mm Whole** | ***0.986*** | **-28.100** | **38.640** |
|  | **1-3mm Whole** | ***0.992*** | **-20.020** | **28.170** |
|  | 1-3mm S | *0.988* | -27.760 | 34.550 |
|  | 1-3mm T | *0.988* | -26.530 | 33.820 |
|  | 1-3mm I | *0.987* | -29.820 | 35.710 |
|  | 1-3mm N | *0.993* | -17.460 | 30.170 |
|  | **3-6mm Whole** | ***0.990*** | **-22.870** | **26.740** |
|  | 3-6mm S | *0.960* | -49.850 | 49.030 |
|  | 3-6mm T | *0.995* | -17.070 | 24.510 |
|  | 3-6mm I | *0.993* | -19.100 | 25.240 |
|  | 3-6mm N | *0.977* | -39.630 | 42.280 |
| **Outer Retina** | **0-1mm Whole** | ***0.995*** | **-3.672** | **1.724** |
|  | **1-3mm Whole** | ***0.996*** | **-2.318** | **0.847** |
|  | 1-3mm S | *0.984* | -3.497 | 2.654 |
|  | 1-3mm T | *0.985* | -3.903 | 2.891 |
|  | 1-3mm I | *0.991* | -3.709 | 1.349 |
|  | 1-3mm N | *0.988* | -3.792 | 2.129 |
|  | **3-6mm Whole** | ***0.995*** | **-2.238** | **0.886** |
|  | 3-6mm S | *0.992* | -2.264 | 1.552 |
|  | 3-6mm T | *0.959* | -5.558 | 4.280 |
|  | 3-6mm I | *0.990* | -3.269 | 1.213 |
|  | 3-6mm N | *0.980* | -4.324 | 2.945 |
| **Whole Retina** | **0-1mm Whole** | ***0.998*** | **-3.580** | **1.850** |
|  | **1-3mm Whole** | ***0.998*** | **-2.684** | **0.672** |
|  | 1-3mm S | *0.997* | -3.012 | 1.468 |
|  | 1-3mm T | *0.994* | -4.527 | 2.165 |
|  | 1-3mm I | *0.996* | -3.577 | 1.437 |
|  | 1-3mm N | *0.998* | -3.083 | 1.074 |
|  | **3-6mm Whole** | ***0.998*** | **-2.793** | **0.884** |
|  | 3-6mm S | *0.996* | -3.188 | 1.692 |
|  | 3-6mm T | *0.983* | -6.336 | 4.063 |
|  | 3-6mm I | *0.995* | -3.918 | 1.954 |
|  | 3-6mm N | *0.987* | -5.825 | 3.878 |
| **RNFL** | **0-1mm Whole** | **0.709** | **-1.985** | **1.531** |
|  | **1-3mm Whole** | ***0.961*** | **-1.248** | **1.180** |
|  | 1-3mm S | *0.959* | -1.857 | 1.911 |
|  | 1-3mm T | 0.856 | -1.672 | 1.150 |
|  | 1-3mm I | *0.941* | -1.944 | 1.871 |
|  | 1-3mm N | *0.950* | -1.292 | 1.553 |
|  | **3-6mm Whole** | ***0.989*** | **-1.940** | **1.777** |
|  | 3-6mm S | *0.990* | -1.918 | 2.101 |
|  | 3-6mm T | 0.822 | -3.921 | 3.060 |
|  | 3-6mm I | *0.989* | -2.733 | 2.448 |
|  | 3-6mm N | *0.985* | -3.196 | 3.452 |
| **GCC** | **0-1mm Whole** | ***0.997*** | **-1.166** | **1.364** |
|  | **1-3mm Whole** | ***0.998*** | **-1.244** | **0.788** |
|  | 1-3mm S | *0.992* | -2.170 | 2.020 |
|  | 1-3mm T | *0.994* | -2.156 | 1.199 |
|  | 1-3mm I | *0.990* | -2.349 | 1.954 |
|  | 1-3mm N | *0.995* | -1.652 | 1.323 |
|  | **3-6mm Whole** | ***0.996*** | **-1.616** | **1.421** |
|  | 3-6mm S | *0.994* | -2.056 | 1.917 |
|  | 3-6mm T | *0.985* | -2.592 | 2.161 |
|  | 3-6mm I | *0.991* | -2.521 | 2.625 |
|  | 3-6mm N | *0.990* | -3.122 | 2.775 |

S, superior; T, temporal; I, inferior; N, nasal; ICC, Intraclass correlation coefficient. Values were analyzed by ICC and Bland-Altman approach. Italic values indicate significance at *P*<0.05 and ICC>0.900. Parameters commonly used are in bold.

Supplemental table 15. Agreement analysis of thickness of choroid, outer retina, whole retina, retinal nerve fiber layer (RNFL), and the ganglion cell complex (GCC) in 4.5×4.5mm scan before and after instillation of tropicamide/phenylephrine mixture eye drops.

| Layer | Location | *ICC* | 95% Limits of Agreement | |
| --- | --- | --- | --- | --- |
|  |  |  | From | To |
| **Choroid** | **0-2mm Whole** | ***0.992*** | **-3.813** | **8.512** |
|  | **2-4mm Whole** | ***0.992*** | **-8.964** | **14.470** |
|  | 2-4mm S | *0.984* | -14.480 | 19.810 |
|  | 2-4mm I | *0.996* | -6.547 | 12.270 |
|  | 2-4mm NS | *0.984* | -15.170 | 20.290 |
|  | 2-4mm NI | *0.993* | -8.903 | 14.170 |
|  | 2-4mm IN | *0.993* | -8.957 | 14.280 |
|  | 2-4mm IT | *0.994* | -8.502 | 14.760 |
|  | 2-4mm TI | *0.997* | -6.628 | 13.020 |
|  | 2-4mm TS | *0.993* | -10.990 | 15.840 |
|  | 2-4mm ST | *0.984* | -15.110 | 22.350 |
|  | 2-4mm SN | *0.966* | -24.960 | 29.480 |
| **Outer Retina** | **0-2mm Whole** | ***0.983*** | **-5.356** | **4.924** |
|  | **2-4mm Whole** | ***0.989*** | **-2.696** | **2.337** |
|  | 2-4mm S | *0.964* | -5.786 | 5.924 |
|  | 2-4mm I | *0.980* | -4.060 | 3.148 |
|  | 2-4mm NS | *0.983* | -2.872 | 2.057 |
|  | 2-4mm NI | *0.983* | -2.856 | 2.541 |
|  | 2-4mm IN | *0.987* | -3.694 | 2.032 |
|  | 2-4mm IT | *0.968* | -7.545 | 6.211 |
|  | 2-4mm TI | *0.972* | -8.916 | 8.565 |
|  | 2-4mm TS | *0.972* | -7.446 | 9.926 |
|  | 2-4mm ST | *0.929* | -13.060 | 14.530 |
|  | 2-4mm SN | 0.870 | -14.920 | 12.930 |
| **Whole Retina** | **0-2mm Whole** | ***0.992*** | **-23.160** | **20.250** |
|  | **2-4mm Whole** | ***0.992*** | **-5.987** | **4.411** |
|  | 2-4mm S | *0.975* | -11.340 | 9.881 |
|  | 2-4mm I | *0.988* | -7.469 | 5.841 |
|  | 2-4mm NS | *0.975* | -12.420 | 13.030 |
|  | 2-4mm NI | *0.988* | -7.717 | 8.398 |
|  | 2-4mm IN | *0.980* | -14.790 | 13.790 |
|  | 2-4mm IT | *0.958* | -21.270 | 18.900 |
|  | 2-4mm TI | *0.961* | -16.830 | 11.940 |
|  | 2-4mm TS | *0.954* | -13.080 | 13.790 |
|  | 2-4mm ST | *0.934* | -28.880 | 21.600 |
|  | 2-4mm SN | *0.902* | -39.050 | 37.220 |
| **RNFL** | **0-2mm Whole** | ***0.986*** | **-27.710** | **24.660** |
|  | **2-4mm Whole** | ***0.986*** | **-6.311** | **5.239** |
|  | 2-4mm S | *0.945* | -17.350 | 15.590 |
|  | 2-4mm I | *0.966* | -10.330 | 10.120 |
|  | 2-4mm NS | *0.978* | -10.920 | 11.130 |
|  | 2-4mm NI | *0.988* | -6.957 | 7.283 |
|  | 2-4mm IN | *0.979* | -13.890 | 15.880 |
|  | 2-4mm IT | *0.946* | -28.390 | 28.270 |
|  | 2-4mm TI | *0.989* | -11.430 | 7.575 |
|  | 2-4mm TS | *0.980* | -12.450 | 9.995 |
|  | 2-4mm ST | *0.907* | -45.170 | 37.050 |
|  | 2-4mm SN | 0.860 | -55.250 | 56.560 |
| **GCC** | **0-2mm Whole** | ***0.988*** | **-26.380** | **23.820** |
|  | **2-4mm Whole** | ***0.988*** | **-6.216** | **5.021** |
|  | 2-4mm S | *0.946* | -16.940 | 15.280 |
|  | 2-4mm I | *0.975* | -9.449 | 8.855 |
|  | 2-4mm NS | *0.977* | -11.040 | 12.470 |
|  | 2-4mm NI | *0.990* | -6.304 | 7.152 |
|  | 2-4mm IN | *0.982* | -13.900 | 14.750 |
|  | 2-4mm IT | *0.946* | -27.650 | 26.820 |
|  | 2-4mm TI | *0.984* | -12.010 | 7.790 |
|  | 2-4mm TS | *0.979* | -10.930 | 8.755 |
|  | 2-4mm ST | *0.909* | -43.110 | 33.950 |
|  | 2-4mm SN | 0.867 | -53.250 | 53.840 |

S, superior; I, inferior; NS, nasosuperior; NI, nasoinferior; IN, inferonasal; IT, inferotemporal; TI, temporoinferior; TS, temporosuperior; ST, superotemporal; SN, superonasal; ICC, Intraclass correlation coefficient. Values were analyzed by ICC and Bland-Altman approach. Italic values indicate significance at *P*<0.05 and ICC>0.900. Parameters commonly used are in bold.
